# Intake of Natural Compounds and Circulating microRNA Expression Levels: Their Relationship Investigated in Healthy Subjects With Different Dietary Habits

**DOI:** 10.3389/fphar.2020.619200

**Published:** 2021-01-14

**Authors:** Giulio Ferrero, Sara Carpi, Beatrice Polini, Barbara Pardini, Paola Nieri, Alessia Impeduglia, Sara Grioni, Sonia Tarallo, Alessio Naccarati

**Affiliations:** ^1^ Department of Clinical and Biological Sciences, University of Turin, Torino, Italy; ^2^ Department of Computer Science, University of Turin, Torino, Italy; ^3^ Department of Pharmacy, University of Pisa, Pisa, Italy; ^4^ NEST, Istituto Nanoscienze-CNR and Scuola Normale Superiore, Pisa, Italy; ^5^ Italian Institute for Genomic Medicine (IIGM), c/o IRCCS Candiolo, Torino, Italy; ^6^ Candiolo Cancer Institute, FPO-IRCCS, Torino, Italy; ^7^ Epidemiology and Prevention Unit, Fondazione IRCCS Istituto Nazionale dei Tumori di Milano, Milan, Italy

**Keywords:** circulating miRNA, dietary natural compounds, plasma metabolites, vitamin D, vitamin E, sodium, dietary habits

## Abstract

Diet has a strong influence on many physiological processes, which in turn have important implications on a variety of pathological conditions. In this respect, microRNAs (miRNAs), a class of small non-coding RNAs playing a relevant epigenetic role in controlling gene expression, may represent mediators between the dietary intake and the healthy status. Despite great advances in the field of nutri-epigenomics, it remains unclear how miRNA expression is modulated by the diet and, specifically, the intake of specific nutrients. We investigated the whole circulating miRNome by small RNA-sequencing performed on plasma samples of 120 healthy volunteers with different dietary habits (vegans, vegetarians, and omnivores). Dietary intakes of specific nutrients were estimated for each subject from the information reported in the food-frequency questionnaire previously validated in the EPIC study. We focused hereby on the intake of 23 natural compounds (NCs) of the classes of lipids, micro-elements, and vitamins. We identified 78 significant correlations (rho > 0.300, *p*-value < 0.05) among the estimated daily intake of 13 NCs and the expression levels of 58 plasma miRNAs. Overall, vitamin D, sodium, and vitamin E correlated with the largest number of miRNAs. All the identified correlations were consistent among the three dietary groups and 22 of them were confirmed as significant (*p*-value < 0.05) by age-, gender-, and body-mass index-adjusted Generalized Linear regression Model analysis. miR-23a-3p expression levels were related with different NCs including a significant positive correlation with sodium (rho = 0.377) and significant negative correlations with lipid-related NCs and vitamin E. Conversely, the estimated intake of vitamin D was negatively correlated with the expression of the highest number of circulating miRNAs, particularly miR-1277-5p (rho = −0.393) and miR-144-3p (rho = −0.393). Functional analysis of the targets of sodium intake-correlated miRNAs highlighted terms related to cardiac development. A similar approach on targets of those miRNAs correlated with vitamin D intake showed an enrichment in genes involved in hormone metabolisms, while the response to chronic inflammation was among the top enriched processes involving targets of miRNAs negatively related with vitamin E intake. Our findings show that nutrients through the habitual diet influence circulating miRNA profiles and highlight that this aspect must be considered in the nutri-epigenomic research.

## Introduction

Dietary habits play a pivotal role in the maintenance of a healthy status and, thus, in the development of several chronic disorders such as diabetes, cardiovascular diseases, hypertension, metabolic syndrome, osteoporosis, and other disease conditions (Ortega et al., 2016; Schulze et al., 2018). It has been well established that Western diet, characterized by high intakes of high-fat food, red meat, refined grains, and sugary desserts increases intestinal mucosal inflammation (Poullis et al., 2004). Conversely, the Mediterranean diet is recognized as one of the healthiest diets mainly thanks to the large use of extra-virgin olive oil, vegetables, and fruits (Martinez-Gonzalez et al., 2015). Furthermore, it has been reported that the adoption of a vegan/vegetarian diet is associated with a decreased risk of cardiovascular diseases (Kim et al., 2019; Molina-Montes et al., 2020), but also with an increase in haematological diseases (Mcevoy et al., 2012).

Beneficial and harmful effects of different dietary habits on healthy conditions have been largely established clinically in epidemiological studies (Schulze et al., 2018). Nevertheless, the knowledge of the exact mechanisms of action of nutritional compounds has been investigated and reported mainly in cell cultures and animal models and their understanding is not yet complete (Serrano et al., 2015).

Accumulating evidence shows that dietary molecules can alter the epigenetic machinery by direct or indirect interactions with enzymes responsible for DNA methylation and histone modifications, as well as with non-coding RNAs (Ideraabdullah and Zeisel, 2018). Among non-coding RNAs, microRNAs (miRNAs) are small non-coding molecules that control fundamental biological processes through the regulation of gene expression mostly at the post-transcriptional level (Briskin et al., 2020). Based on the current knowledge, it is important to evaluate which dietary components can control and perturb miRNAs involved in the regulation of long-term physiological health and pathological processes.

Several bioactive nutritional factors have been found to modulate miRNA expression levels both in human studies and *in vitro* (Quintanilha et al., 2017). For instance, nine serum miRNAs were modulated in young male subjects in response to dietary zinc depletion/repletion and these miRNAs were also associated with inflammation (Ryu et al., 2011). In a human intervention study with supplementation of 25-hydroxyvitamin D in males, serum concentrations of this vitamin showed a weak correlation to circulating miR-532-3p (Jorde et al., 2012). An interesting recent article highlighted that circulating levels of specific miRNAs could predict the most successful dietary model for preventing the development of type 2 diabetes mellitus in coronary heart disease patients. In detail, low baseline plasma levels of miR-145 were associated with a higher risk of developing type 2 diabetes mellitus in those subjects consuming a low-fat high-complex carbohydrate diet (Jimenez-Lucena et al., 2020). Polyphenols of extra-virgin olive oil may control *in vitro* adipocytes inflammation through the modulation of miR-155, let-7c and miR-34a (Carpi et al., 2019; Scoditti et al., 2019). Indeed, natural compounds (NCs) occurring in the diet seem able to control and suppress some human pathological conditions such as inflammation, autoimmune response, and cancer via epigenetic regulation of miRNA expression (Ideraabdullah and Zeisel, 2018). Conversely, NCs could promote human diseases by up-regulating the expression of various inflammatory, immune and/or oncogenic miRNAs and thus initiate cellular processes such as inflammation, immune response, and tumor proliferation (Samec et al., 2019).

Furthermore, it is mandatory to investigate the link between natural dietary compounds and miRNA expression levels to develop effective pharmacological agents against deadly diseases. These data could allow a better understanding of the impact of dietary compounds (in single or as clusters) on epigenetic regulation, and finally helping in the selection of novel drug candidates from the plethora of natural molecules. However, although *in vitro* and *in vivo* models have shown the ability of single nutrients to modulate miRNA expression (Ross and Davis, 2014; Biersack, 2016; Kura et al., 2019), data concerning miRNA modulation in the context of different dietary habits in humans are very scarce. In a pilot study, we have previously documented the differences in the expression profiles of circulating miR-16, miR-21, miR-34a, and miR-222 when associated with different dietary habits (Tarallo et al., 2014).

In the present study, we hypothesized that dietary habits may modulate the expression of circulating miRNAs and, through their regulation, to have implications on health. We therefore measured and compared the expression of the whole circulating miRNome in plasma samples from healthy volunteers (n = 120) following different dietary habits (vegan, vegetarian and omnivorous diets). Derived miRNA profiles were related to a daily intake of NCs derived from detailed reported questionnaires filled by all subjects.

## Materials and Methods

### Study Population

A cohort of 120 healthy Italian volunteers, 72 women and 48 men was recruited in the Piedmont region and included an equal proportion of vegetarians, vegans, and omnivores. Vegetarian and vegan volunteers were recruited with the collaboration of the Italian Society of Vegetarian Nutrition (http://www.scienzavegetariana.it) or, as of the omnivores, among personal contacts and by distributing an informative leaflet. The inclusion criteria for the constitution of the cohort were the following: dietary regime followed for more than one year, no use of antibiotics in the previous three months, absence of evidence of intestinal pathologies (Crohn's disease, chronic ulcerative colitis, bacterial overgrowth syndrome, constipation, celiac disease, Irritable Bowel Syndrome), and other pathologies (type I or type II diabetes, cardiovascular or cerebrovascular diseases, cancer, neurodegenerative disease, rheumatoid arthritis, allergies), absence of pregnancy and lactation. Anthropometric data (body mass index (BMI) and abdominal circumference) were collected for all subjects at the moment of the collection of the biological samples (stool and blood). BMI classes were defined based on World Health Organization (WHO) (https://www.who.int/) thresholds: Underweight: BMI<18.5; Healthy weight: 18.5 ≤ BMI≤25; Overweight: 26 ≤ BMI≤30; Obese: BMI>30. All subjects were recruited between May 2017 and July 2019. The design of the study, the informed written consent and protocols were approved by the local Ethics Committee on Colorectal_miRNA CEC 2014 (Azienda Ospedaliera “SS. Antonio e Biagio e C. Arrigo” of Alessandria).

### Estimation of Natural Compound Intake From EPIC Questionnaires

All recruited subjects filled in a validated self-administered food-frequency questionnaire (FFQ) assessing the usual diet, together with lifestyle and personal history data, in accordance with the EPIC study standards (Pala et al., 2003; Vineis and Riboli, 2009). The FFQ consisted of 248 questions concerning 188 different food items and included photos with two or three sample dishes of definite sizes, or references to standard portion sizes. The composition in nutrients of individual food items was obtained from Italian food composition tables (Salvini et al., 1998) and the average intake of macro and micronutrients for each volunteer was estimated. The questionnaires were subsequently filled in on the web platform AcQUE, developed using technologies such as the Node.js platform and the Angular framework. The platform was implemented to create a single digital environment where users can create *ad hoc* questionnaires for specific studies, assemble them in projects and then fill them in AcQUE enabling users to consult subjects’ questions and answers directly on the platform with data exportable in a .csv file format. The following NCs were considered for the analysis: β-carotene, calcium, cholesterol, folic acid, iron, linoleic acid, linolenic acid, monounsaturated fatty acids, niacin, oleic acid, phosphorus, potassium, retinol, riboflavin, sodium, thiamine, total polyunsaturated fatty acids, total saturated fatty acids, vitamin B6, vitamin C, vitamin D, vitamin E, and zinc.

### Sample Collection and Total RNA Extraction From Plasma

Blood samples were collected according to standard phlebotomy procedures at the moment of the recruitment. Samples were collected into ethylenediaminetetraacetic acid (EDTA) tubes and immediately processed for plasma separation with centrifugation at × 1000 g for 10 min at room temperature. Once separated from the rest of blood, plasma was distributed in cryovial tubes. One tube was immediately used for RNA extraction while the other aliquots were labelled and stored at −80°C. The time interval between sample collection, processing and storage at −80°C was less than 3 h.

Total RNA was extracted from 200 µL of plasma with the miRNeasy plasma/serum mini kit (Qiagen) using the QiaCube extractor (Qiagen) following the manufacturer's instructions. RNA samples were stored until analysis at −80°C.

### Small RNA-Sequencing Analysis

Library preparation was performed with the NEBNext Multiplex Small RNA Library Prep Set for Illumina (Protocol E7330, New England BioLabs Inc., USA) as described in (Sabo et al., 2020) and (Ferrero et al., 2018). For each sample, six ul of RNA was used as starting material to prepare libraries. Each library was prepared with a unique indexed primer so that the libraries could all be pooled into one sequencing lane. The obtained sequence libraries (24-samples multiplexed) were subjected to the Illumina sequencing pipeline, passing through clonal cluster generation on a single-read flow cell (Illumina Inc., USA) by bridge amplification on the cBot (TruSeq SR Cluster Kit v3-cBOT-HS, Illumina Inc., USA) and 50 cycles sequencing-by-synthesis on the HiSeq 2000 (Illumina Inc., USA) (in collaboration with Genecore Facility at EMBL, Heidelberg, Germany).

The analysis of sequencing data was performed as described in (Tarallo et al., 2019). Specifically, Fastq files were quality-controlled with FastQC (http://www.bioinformatics.babraham.ac.uk/projects/fastqc). Reads shorter than 14 nucleotides were discarded. The QC-passed reads were clipped from the adapter sequences using Cutadapt v1.10 by imposing a maximum error rate in terms of mismatches, insertions, and deletions equal to 0.15. Trimmed reads were mapped against a miRNA reference based on miRBase v22 annotations (Kozomara et al., 2019) using the small RNA-Seq module of Docker4Seq (Kulkarni et al., 2018). The alignment was performed using BWA algorithm v0.7.127 with the default settings. Using these settings, the seeding was not performed for reads shorter than 32 bp, and the reads were entirely evaluated for the alignment. The human miRNAs were annotated and quantified using two methods called the “knowledge-based” and “position-based” methods as described in Tarallo et al. (2019). miRNAs whose assigned arms were derived from the “position-based” methodology were indicated in italics. The results generated by the annotation and quantification methods were merged into a unique mature miRNA count matrix. In the case of mature miRNAs processed from different precursors but characterized by the same mature sequence, the read counts were summed.

miRNA expression levels were normalized using DESeq2 R package (Love et al., 2014). A miRNA was considered detectable if associated with a normalized number of reads greater than 15.

The raw small RNA-Seq reads and raw read count table were deposited in Gene Expression Omnibus (GEO) with the identifier GSE160130.

### Correlation Analysis Between Plasma miRNAs Expression and Natural Compound Intake

The correlation between the normalized miRNA expression levels and the estimated NC daily intake was computed by considering those miRNAs characterized by a median number of reads greater than 15 in at least a dietary group. The correlation was calculated using the Spearman correlation method implemented in the *rcorr* function of the *Hmisc* R package. The correlation was computed considering each dietary group separately. Only coherent correlations among the three diets (associated with positive or negative coefficients in all dietary groups) and associated with a *p*-value < 0.05 in at least one group were considered for further analysis.

### Generalized Linear Regression Model

The Generalized Linear regression Model (GLM) was computed using the *glm* R function. The miRNA expression level was considered as the dependent variable of the model while subjects’ age, sex, BMI, and estimated daily intake of each NC were considered as the independent variables of the model. The significance of the GLM model was computed with respect to a null-model using F-test implemented in the *anova* R function. Models associated with a *p*-value < 0.05 were considered as statistically significant. The contribution of a NC to the model was considered significant if the associated *p*-value was lower than 0.05 and lower than those computed for the contribution to the model of sex, age, and BMI. The graphical representation of the significant miRNA-NC interactions was performed using Cytoscape v3.8.0 (Shannon et al., 2003). Specifically, miRNAs and the three classes of considered NC (lipid-related, micro-element, vitamin-related) were represented as network nodes using different colors and with node size proportional to the node degree. The network edges were colored based on the values of the Spearman rho, and their thickness was represented as proportional to the absolute correlation value. Furthermore, miRNA-NC pairs were associated with a continuous edge if the association was significant also in the GLM model while dashed edges were used for representing non-significant association in the GLM analysis.

### Differential Expression Analysis and miRNA Targets Enrichment Analysis

The miRNA differential expression (DE) analysis was performed among dietary groups and between groups of subjects divided into high and low intake individuals based on the median daily intake of the NC considered. The DE analysis was performed using DESeq2 v 1.24.0 (Love et al., 2014). The package was applied using the Log-Rank Test (LRT) method and correcting the analysis by age, sex, BMI, and dietary class. A miRNA was considered as differentially expressed if associated with a *p*-value < 0.05 and a median number of reads greater than 15 in at least one subject group. The calculated log2 Fold-change (FC) and adjusted *p*-value from the DE analysis were used as input for the miRNA target functional enrichment analysis. Functional enrichment analysis of miRNA target genes was performed using RBiomirGS v0.2.12 (Zhang and Storey, 2018) and the miRNA-target gene annotations from miRTarbase v7.0 (Chou et al., 2018) and miRecords (Xiao et al., 2009). The analysis was performed on gene set library GO Biological Processes retrieved from the Molecular Signatures Database v7.1 (Liberzon et al., 2011; Liberzon et al., 2015). The analysis was performed separately on miRNAs correlated and anticorrelated with a specific NC. Only enriched processes associated with an adjusted *p*-value < 0.05, an absolute coefficient >0.5, and involving at least two miRNA targets were considered. Data about miRNA expression levels in different human tissues and cell models were retrieved from the FANTOM five project (De Rie et al., 2017). Specifically, the miRNA cell ontology data were downloaded from the project website dedicated to miRNA expression analysis (https://fantom.gsc.riken.jp/5/suppl/De_Rie_et_al_2017/vis_viewer/). These data represent the statistically significant enrichment or depletion of miRNA expression in specific cell ontology clusters as defined in (Meehan et al., 2011).

## Results

### An Overview of the Relationship Between miRNAs Expression Levels and Natural Compound Estimated Intake

**FIGURE 1:**
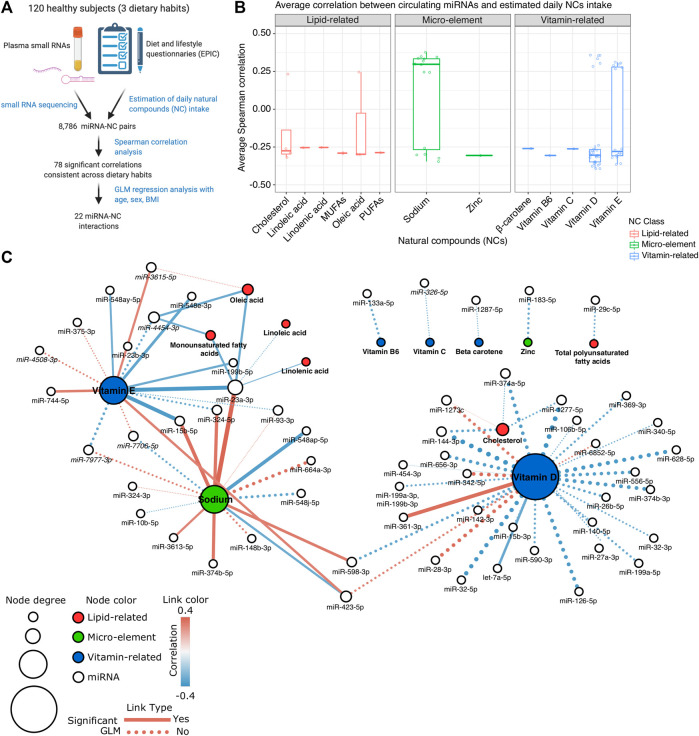
**(A)** Schematic diagram of the study design. **(B)** Box plot showing the average Spearman correlation values computed between miRNA expression levels and the estimated intake of different Natural Compounds (NCs). **(C)** Network representation of the identified significant interactions between miRNAs and NCs. Each white node represents a miRNA; red, green, and blue nodes represent lipid-related, micro-element, or vitamin-related NCs, respectively. The size of the node is proportional to the node total degree. Each edge represents a statistically significant miRNA-NC correlation (*p*-value < 0.05 in at least two dietary groups). The correlation value is represented as the edge color while the edge width represents the absolute correlation coefficient. Dotted edges represent the miRNA-NC interactions significant also in the GLM analysis.

We investigated the potential relationship between circulating miRNA expression levels and estimated daily NC intake of 120 Italian subjects grouped for their different diets: omnivorous, vegetarian, and vegan (Figure 1A). Circulating miRNA levels were assessed by small RNA-seq of RNA isolated from plasma samples while nutrient intake was estimated from FFQ assessing the usual diet in accordance with the EPIC study standards. Among the three dietary groups no significant differences were observed in terms of gender (chi-square *p*-value > 0.05) and age (Kruskal-Wallis *p*-value > 0.05) distributions. In contrast, BMI was higher in omnivorous subjects compared to vegetarians and vegans (Wilcoxon Rank-Sum test *p*-value < 0.05) (Supplementary Table S1). Conversely, no significant differences were observed among the distribution of BMI classes (healthy weight, underweight, overweight, obese) among the three dietary groups.

On average, 30.7% of the reads obtained from small RNA-seq aligned on miRNA sequences and an average of 348 miRNAs were detected in each sample (Supplementary Table S2A). No significant differences were observed between plasmatic miRNA expression profiles among the three dietary groups (Supplementary Table S2B).

Using Spearman correlation analysis, we tested 8,786 miRNA-NC combinations involving the expression levels of 382 miRNAs detectable in plasma samples and the estimated Kcal-normalized daily intake of 23 NCs (Supplementary Table S3A). Among these combinations, 2,534 were characterized by a coherent correlation/anticorrelation among the three diet-specific groups of subjects recruited (Supplementary Table S3A). Of all these last coherent correlations, 78 were statistically significant (absolute Spearman rho >0.3 and *p*-value < 0.05 in at least two diet groups) and involved in total 58 miRNAs and 13 NCs (Figure 1B; Supplementary Table S3B). Among the investigated 13 NCs, vitamin D, sodium, and vitamin E correlated with the highest number of miRNAs (n = 30, 16, and 16, respectively). Conversely, miR-23a-3p, followed by miR-423-5p, and *miR-4454-3p* were significantly correlated with the highest number of different NCs (n = 6, 3, and 3, respectively).

To identify those miRNAs recurrently correlated within specific NC classes, we grouped all NCs into three major classes: lipid-related, vitamin-related, and micro-elements (Figures 1B,C). Among the lipid-related NCs, 12 significant correlations were observed. The strongest correlation resulted between cholesterol intake and decreased levels of miR-144-3p (average rho = −0.319), particularly in vegans (rho = −0.452, *p*-value < 0.01), and vegetarians (rho = −0.323, *p*-value < 0.05). Cholesterol was also the lipid-related NC characterized by the highest number of related miRNAs (n = 4). miR-23a-3p was correlated to the highest number of NCs of the lipid-related group (n = 4, average rho ranging from −0.253 to −0.299). A total of 49 significant correlations were observed among vitamin-related NCs and miRNA profiles. Vitamin D showed the strongest inverse correlation with miR-1277-5p (average rho = −0.393) and the most significant positive one with miR-342-5p (average rho = 0.358) (Supplementary Table S3B; Figure 1C). As represented in Figure 1C, four vitamin D-correlated miRNAs (miR-144-3p, miR-1273c, miR-374a-5p, and miR-1277-5p) were also coherently correlated with the cholesterol intake. Finally, 17 significant correlations were observed considering the micro-element NC group and the most significant correlation emerged between sodium intake and miR-23a-3p expression levels (average rho = 0.377). Differently from the other NC groups in which negative correlations prevailed, most of the correlations with sodium (n = 10, 62.5%) were positive (Figure 1C).

**FIGURE 2:**
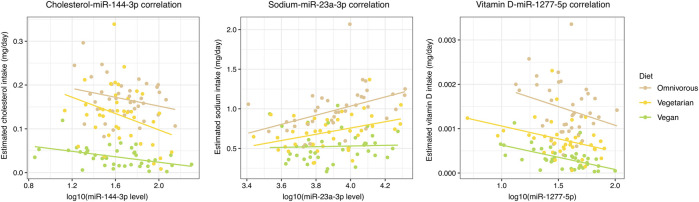
Scatterplot reporting the most significant NC-miRNA correlations for each NC class with a progressive increment or decrement in absolute correlation value in the three dietary groups. A color-code is used to distinguish subjects from each dietary group and the corresponding linear regression line is reported.

Among the 78 significant NC-miRNA correlations, 40 (51.3%) were associated with absolute values that progressively increase or decrease among the three dietary groups (Figure 2; Supplementary Table S3). Specifically, 21 correlations increased progressively from omnivores to vegans and they involved mainly vitamin D (17 correlations) and cholesterol (four correlations). Conversely, the remaining 19 NC-miRNA correlations progressively decreased from omnivores to vegans. These correlations involved mainly sodium and vitamin E (seven correlations for each NC).

Finally, we performed a GLM analysis to explore the miRNA expression profiles in our study population considering each NC intake and the individual age, sex, and BMI contribution. Among the previously reported 78 significant miRNA-NC correlations, 31 (39.7%) were characterized by a significant general GLM model (*p*-value < 0.05) while for 22 combinations, the estimated daily NC intake represented the most significant contribution to the model (*p*-value < 0.05) (Supplementary Table S4 and dashed edges on Figure 1C). The most significant GLM model considering the lipid-related NCs was between monounsaturated fatty acids and miR-23a-3p (*p*-value < 0.01) while the combination between sodium and miR-148b-3p (*p*-value < 0.0001), and between vitamin E and *miR-7977-3p* (*p*-value < 0.0001) were the most significant combinations observed considering the micro-element and the vitamin-related group, respectively (Supplementary Table S4).

### Differential Expression and Enrichment Analysis

Focusing only on NCs characterized by the highest number of significant correlations with miRNA expression levels (i.e., vitamin D, sodium, vitamin E, and cholesterol), we subsequently stratified our study population according to a high/low median daily intake of each specific compound and we compared miRNA profiles between these subgroups. As reported in Supplementary Table S5, 26 miRNAs out of the 30 related with vitamin D, 13 miRNAs out of the 16 correlated with sodium, and four miRNAs out of the 16 related with vitamin D intake were also characterized by distinct significantly different expression levels (*p*-value < 0.05) between individuals with high and low intake of each NC (Supplementary Table S6A). As expected, the log2FC of the DE analysis was coherent with the result of the correlation analysis, with NC positively correlated miRNAs upregulated in high intake individuals and vice versa. In particular, the most significant result was obtained for the vitamin D-correlated miR-342-5p which was significantly upregulated in individuals with the highest intake of this vitamin (log2FC = 0.78, adjusted *p*-value < 0.01). Conversely, in individuals who reported a high sodium intake, a significant downregulation of miR-423-5p was observed (log2FC = −0.45, adjusted *p*-value < 0.05) coherently with the significant negative relation of this miRNA with the NC (rho = −0.30).

**FIGURE 3:**
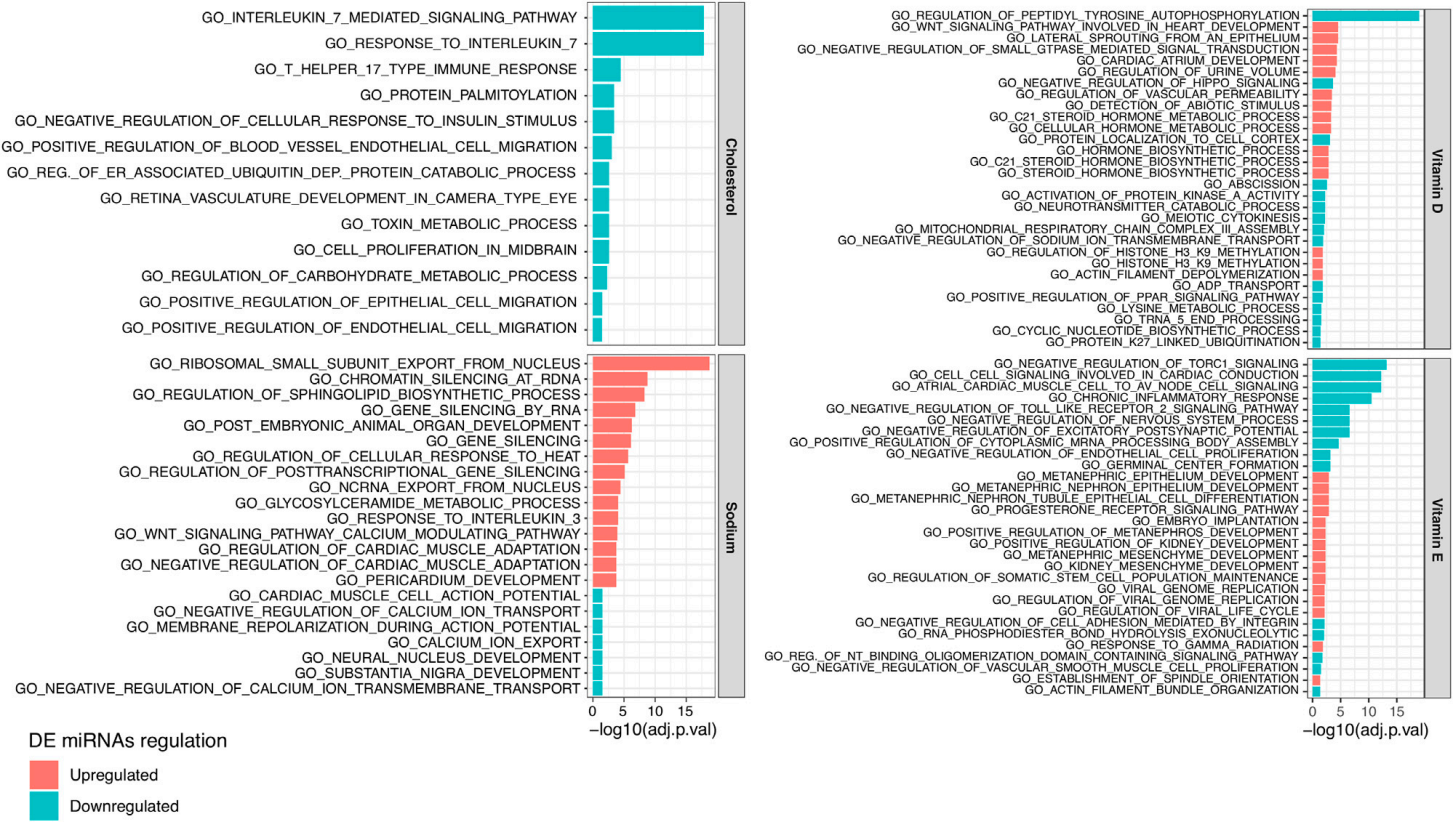
Bar plot reporting the 15 top significant Gene Ontology (GO) Biological Processes enriched in the target genes of plasmatic miRNAs resulting downregulated (in blue) or upregulated (in red) between subjects characterized by a higher or lower estimated intake of (from the top left corner in clockwise order) cholesterol, vitamin D, sodium, or vitamin E.

Based on the generated log2FC and significance of each DE miRNA, we performed a functional enrichment analysis of the biological processes involving miRNA targets. In total, we observed 102, 13, 42, and 30 biological processes significantly enriched (adjusted *p*-value < 0.05) in targets of miRNAs related to sodium, cholesterol, vitamin D, and vitamin E, respectively (Supplementary Table S6B). Figure 3 reports the most significantly enriched processes for each NC. Considering those miRNAs positively related to sodium, their targets were prevalently enriched in processes related to gene silencing (e.g., GO_GENE_SILENCING_BY_RNA) while targets of miRNA negatively related to sodium were mainly enriched in terms related to ion transport (e.g., GO_CALCIUM_ION_EXPORT). The targets of cholesterol negatively related miRNAs were enriched in terms connected to Interleukin-17 response (e.g., GO_INTERLEUKIN_7_MEDIATED_SIGNALING_PATHWAY) and endothelial cell migration (e.g., GO_POSITIVE_REGULATION_OF_BLOOD_VESSEL_ENDOTHELIAL_CELL_MIGRATION) while different terms related to steroid hormone metabolic processes (e.g., GO_C21_STEROID_HORMONE_METABOLIC_PROCESS) were enriched in targets of vitamin D related miRNAs. Finally, targets of vitamin E related miRNAs were enriched in heterogeneous terms, including GO_CHRONIC_INFLAMMATORY_RESPONSE.

## Discussion

The interaction between diet and miRNA expression has been only recently explored. Although the potential of exogenous diet-derived miRNAs (also called dietary miRNAs) is characterized by contradictory results, the ability of diet-derived NCs to modulate miRNA expression is supported by growing evidence (Quintanilha et al., 2017).

Here, we demonstrated that NC intake may affect the expression levels of several circulating miRNAs in humans, independently of their different dietary regimes. In detail, we identified 78 NC-miRNA relations consistent among the three different dietary groups. Among them, 22 were also supported by a GLM analysis including main covariates potentially affecting miRNA expression levels: age, gender, and BMI. Among all the analysed NCs, sodium, cholesterol, vitamin D, and vitamin E displayed the highest number of correlations with miRNA expression profiles.

Sodium is an essential nutrient, required for normal physiological functions, and the main source of its intake from diet is represented by salt (sodium chloride) (Kotchen et al., 2013). Hereby, we observed that sodium intake was associated with altered levels of 16 plasma miRNAs. More specifically, the circulating miRNA that increased most significantly in relation to sodium intake was miR-23a-3p, a miRNA abundant in both plasma and extracellular vesicles (Freedman et al., 2016; Godoy et al., 2018) and whose expression is enriched in “endothelial cells of vascular tree”, as evicted from the analysis of FANTOM 5 expression data (De Rie et al., 2017) (Supplementary Table S7). Evidence reported its deregulation in many cardiovascular diseases (Feldman et al., 2017). Increased expression of miR-23a-3p can also predict the presence of coronary lesions (Di et al., 2015), and altered circulating levels of this miRNA were reported in postoperative atrial fibrillation development (Feldman et al., 2017). Furthermore, miR-23a-3p has been involved in cardiomyocyte growth: NFATc3 (nuclear of activated T-cells) directly induces miR-23a-3p expression, which in turn inhibits the translation of muscle-specific ring finger protein 1, an anti-hypertrophic protein (Lin et al., 2009). This miRNA is also involved in the regulation of apoptosis in cardiomyocytes by targeting manganese superoxide dismutase (*MnSOD*) (Long et al., 2017). In agreement with these observations and the reported role of miR-23a-3p, the sodium-associated miRNAs appeared able to regulate many genes involved in biological processes strongly related to cardiac function (GO_REGULATION_OF_CARDIAC_MUSCLE_ADAPTATION, GO_CALCIUM_ION_EXPORT, and GO_CARDIAC_MUSCLE_CELL_ACTION_POTENTIAL) (Ottolia et al., 2013). Since the relationship between sodium levels, blood pressure and cardiovascular diseases is well-known (O’donnell et al., 2010), the ability of sodium to regulate the expression of miRNAs, such as miR-23a-3p can contribute to explain the strong correlation between this NC and cardiac functions.

Among the lipid-related NCs, cholesterol showed the highest number of miRNAs inversely correlated to its intake (miR-144-3p, miR-374a-5p, miR-1277-5p and miR-1273c). miR-144-3p is the most significant inversely related with cholesterol. This miRNA targets the ATP-binding cassette transporter A1 (*ABCA1*), which is known for increasing atherosclerotic burden and pro-inflammatory cytokines (De Aguiar Vallim et al., 2013; Hu et al., 2014). Interestingly, the circulating level of this miRNA was significantly increased in elderly subjects characterized by a decreased circulating fatty acids levels after a one-year walnuts enriched diet (Lopez De Las Hazas et al., 2020). Another miRNA negatively correlated with cholesterol, miR-374a-5p, is able to regulate the expression of many target genes involved in carbohydrates and lipids biosynthesis and inflammation, particularly interleukin-17A, in turn, involved in lipid metabolism (Yu et al., 2014; Doumatey et al., 2018). Also, miR-1277-5p and miR-1273c have been reported as altered in inflammatory statuses (Zalewski et al., 2020). Altogether, the observed anti-correlations between cholesterol intake and these miRNAs suggest that cholesterol itself could participate in the regulation of its efflux in macrophages, as recently reported for HDL (Orekhov et al., 2018).

Noteworthy, the same cholesterol-correlated miRNAs were also correlated with vitamin D. The connection between these 2 NCs is not surprising if we consider the involvement of vitamin D in lipid metabolism and inflammation (Ning et al., 2015). In our analysis, vitamin D was characterized by the highest number of related miRNAs with most of them inversely related to an estimated intake of this NC. One of the most significant miRNAs negatively related to vitamin D was miR-144-3p, which is highly expressed in plasma samples (as reported by (Godoy et al., 2018) and highly abundant in vein tissues as reported by the human miRNA tissue atlas (Ludwig et al., 2016). Interestingly, serum levels of miR-144-3p resulted altered in patients with osteoporosis, and it has been demonstrated that this miRNA regulates osteoclastogenesis by targeting the Receptor activator of nuclear factor κ B (*RANK*) mRNA (Wang et al., 2018). However, establishing a relationship between vitamin D and miR-144-3p in relation to osteoporotic phenotype deserves further investigations. Finally, another miRNA whose circulating levels significantly decreased with the increased intake of vitamin D was let-7a-5p. The result is coherent with the study of Beckett and colleagues. Authors demonstrated a negative correlation between vitamin D intake and circulating levels of let-7a-5p and let-7b-5p and that this correlation was related to specific polymorphisms at the vitamin D receptor (*VDR*) coding gene (Beckett et al., 2014). To the best of our knowledge, only a limited number of studies have explored the relationship between vitamin E intake and miRNA expression levels so far. Gaedicke and colleagues reported that a 6-months diet deficient in vitamin E led to a miRNA dysregulation in rat liver (Gaedicke et al., 2008). On the other hand, Zhang et al., identified miRNAs differentially expressed in chicken adipose tissues before or after a vitamin E enriched diet (Zhang et al., 2020). Coherently with the known activity of vitamin E in the regulation of inflammation (Lewis et al., 2019), “chronic inflammatory response” was one of the most significant functional terms in this miRNA target enrichment analysis. Three genes annotated in this functional term were validated targets of miR-23a-3p (Supplementary Table S6C), whose levels were inversely related to vitamin E in our analysis. Specifically, these three genes encode for Gap Junction Protein Alpha 1 (*GJA1*), Tumor Necrosis Factor (TNF) Alpha Induced Protein 3 (*TNFAIP3*), and Vascular Cell Adhesion Molecule 1 (*VCAM1*). Gap junction proteins have a pivotal role in the modulation of the inflammatory response (Wong et al., 2017) and *GJA1* 3′UTR was demonstrated to be a direct target of miR-23a-3p in an osteosarcoma cell model (Gindin et al., 2015). TNFAIP3, also known as A20, is involved in the cytokine-mediated immune response downstream of the TNF signaling pathway (Das et al., 2018). In mouse macrophages, it was demonstrated a direct regulation of this gene by miR-23a-3p (Peng et al., 2015). Despite further experimental evidence being needed to validate a functional relationship between these molecules, our findings are consistent with the established role of circulating miRNAs as mediators of inflammation and immune response (Nejad et al., 2018).

This study represents the first investigation aimed at exploring, at the whole miRNome level, the relationship between circulating human miRNAs and the daily intake of dietary NCs. The subjects in our cohort were carefully recruited to represent an age- and gender-homogenous population in which the dietary habit was the main discriminating factor. Interestingly, the DE analysis among the three dietary groups did not reveal any significant dysregulation in circulating miRNA levels while a subject stratification based on the intake of specific NC did. This is also consistent with the lack of significant differences observed in circulating miRNAs levels among the three dietary groups in our study, despite a significant difference in BMI. Other biospecimens could better reflect the nutri-epigenomic effect of different dietary habits and BMI, particularly stool samples, as previously reported in (Tarallo et al., 2014).

Our results should be carefully interpreted since the significant correlations between circulating miRNAs and NC intake have to be further validated to exclude any potential confounding factors. Moreover, despite GLM analysis was able to confirm a non-significant influence of subject age, sex, and BMI in a subset of correlations, other not investigated covariates could influence the observed miRNA-NC relations. This is quite relevant, since circulating miRNA expression levels can be extensively influenced by different lifestyle habits or other environmental factors (Ameling et al., 2015; Keller et al., 2017; Rounge et al., 2018).

Another aspect to be considered in interpreting our results is that the daily NC intake was not directly measured in plasma samples but estimated from individual FFQ information. Despite, there is a wide agreement on reliability on NC levels estimated from FFQ (Cade et al., 2004; Garcia-Larsen et al., 2011; Beckett et al., 2014; Weir et al., 2016; Vilela et al., 2019), each hematic NC level can be differently influenced by independent host and environment factors, and we cannot exclude the presence of compiling errors or unprecise reporting in the FFQ.

Despite this, our results support the hypothesis of a regulation of circulating miRNA levels tightly related to specific NCs, which can be assumed in different dietary regimens. An intervention study based on a controlled intake of a specific NC is needed to further support our results and to provide novel evidence on the regulatory effect of nutrition on the human circulating transcriptome.

## Data Availability Statement

The datasets presented in this study can be found in online repositories. The names of the repository/repositories and accession number(s) can be found below: Gene Expression Omnibus (GEO identifier = GSE160130).

## Author Contributions

GF, SC, ST, BaP, and AN conceived the study. ST, BaP, and AN, performed the sample collection and wet-lab analyses. GF performed the statistical/computational analyses. AI developed AcQUE database and processed questionnaire data. SG performed the nutrient quantification and conversion from questionnaires. GF, SC, ST, BaP, BeP, PN, SG, and AN interpreted the results and wrote the manuscript. All the authors wrote and approved the manuscript.

## Funding

This work was supported by Compagnia di San Paolo (to AN, BaP, and ST); the Oncobiome European H2020 research project (Grant number 825410) (to AN, BaP, and ST), Lega Italiana per la Lotta contro Tumori (LILT) (to AN, BaP, ST, and GF), Fondazione Umberto Veronesi postdoctoral fellowships 2017 and 2018 (recipients, BaP and ST), Fondazione CRT (grant number 2019-0450) (to AI) and partially supported by University of Pisa funds (to SC and PN).

## Conflict of Interest

The authors declare that the research was conducted in the absence of any commercial or financial relationships that could be construed as a potential conflict of interest.
